# The Mechanisms and Application Prospects of Astrocyte Reprogramming into Neurons in Central Nervous System Diseases

**DOI:** 10.2174/011570159X379061250415094751

**Published:** 2025-05-08

**Authors:** Rongxing Qin, Xinyu Lai, Wei Xu, Qingchun Qin, Xiaojun Liang, Minshan Xie, Li Chen

**Affiliations:** 1 Department of Neurology, The First Affiliated Hospital of Guangxi Medical University, Nanning, Guangxi Zhuang Autonomous Region, 530021, China;; 2 Collaborative Innovation Centre of Regenerative Medicine and Medical BioResource Development and Application, Co-constructed by the Province and Ministry, Guangxi Medical University, Nanning, Guangxi Zhuang Autonomous Region, China;; 3 National Center for International Research of Biological Targeting Diagnosis and Therapy (Guangxi Key Laboratory of Biological Targeting Diagnosis and Therapy Research), Guangxi Medical University, Nanning, Guangxi Zhuang Autonomous Region, 530021, China

**Keywords:** Astrocyte reprogramming, CNS diseases, induced neurons (iNs), MicroRNAs, neurological recovery, small molecules, transcription factors

## Abstract

Central nervous system (CNS) diseases, including ischemic stroke (IS), Alzheimer’s disease (AD), and Parkinson’s disease (PD), are leading causes of global disability and mortality, characterized by progressive neuronal loss and irreversible neural circuit damage. Despite advances in understanding their pathophysiology, therapeutic options remain limited due to the complexity of disease mechanisms and challenges in delivering treatments across the blood-brain barrier (BBB). In this context, astrocyte reprogramming has emerged as a groundbreaking strategy for neural repair. By leveraging the plasticity of astrocytes, researchers have demonstrated the potential to convert these glial cells into functional neurons, offering a novel approach to replenish lost neurons and restore neural function. This review explores the latest advancements in astrocyte reprogramming, focusing on transcription factor-mediated, miRNA-induced, and small molecule-based strategies, as well as the molecular mechanisms underlying this process. We also discuss the therapeutic potential of astrocyte reprogramming in CNS diseases, including IS, AD, PD, and other neurodegenerative disorders, while addressing the challenges and future directions for clinical translation. Through a systematic analysis of recent studies, this review highlights the promise of astrocyte reprogramming as a transformative therapeutic strategy for CNS repair, providing new hope for patients with debilitating neurological conditions.

## 1 INTRODUCTION

Central nervous system (CNS) diseases, affecting approximately 17% of the global population, represent a significant cause of disability, mortality, and socioeconomic burden worldwide. These disorders, including ischemic stroke (IS), Alzheimer’s disease (AD), and Parkinson's disease (PD), are characterized by progressive neuronal loss and irreversible damage to neural circuits [1-4]. IS, for instance, triggers rapid neuronal death due to ischemia-induced damage and subsequent inflammatory cascades, leading to long-term functional deficits [5]. Similarly, AD is driven by the accumulation of amyloid plaques and neurofibrillary tangles, which initiate pathogenic cascades resulting in synaptic loss, neuronal death, and cognitive decline [6]. PD, the second most common neurodegenerative disorder, is marked by the progressive degeneration of dopaminergic neurons in the substantia nigra, leading to motor and non-motor symptoms that worsen with disease progression [7].

Despite advances in understanding the pathophysiology of these diseases, therapeutic interventions remain limited, largely due to the complexity of their underlying mechanisms and the challenges of delivering effective treatments across the blood-brain barrier (BBB) [8, 9]. For example, thrombolysis, the primary treatment for IS, is constrained by a narrow therapeutic window and the risk of hemorrhagic complications [10]. In AD, although anti-amyloid therapies have shown promise, their clinical efficacy remains suboptimal due to the multifactorial nature of the disease [6]. Similarly, in PD, while dopaminergic therapies provide symptomatic relief in the early stages, they fail to address the progressive neurodegeneration and non-dopaminergic features that emerge as the disease advances [11]. These challenges underscore the urgent need for innovative therapeutic approaches that can not only mitigate symptoms but also promote neural repair and functional recovery.

In this context, astrocyte reprogramming has emerged as a promising strategy. By leveraging the plasticity of astrocytes, researchers have demonstrated the potential to convert these glial cells into functional neurons [12, 13]. For instance, overexpression of the transcription factor *NeuroD1* in IS models has been shown to restore approximately one-third of damaged neurons through glial cell-mediated regeneration [14]. Similarly, in PD models, the transdifferentiation of astrocytes into dopaminergic neurons *via* transcriptional regulation offers a potential avenue for halting disease progression [15]. In AD, reprogramming reactive astrocytes into glutamatergic neurons has shown promise in mitigating neuronal and synaptic loss [13]. These findings highlight the transformative potential of astrocyte reprogramming as a therapeutic approach for CNS diseases, offering new hope for neural repair and functional recovery. This review explores the latest advancements in astrocyte reprogramming and its implications for the treatment of CNS diseases.

## 2 SEARCH STRATEGY AND SELECTION CRITERIA

References were systematically retrieved from Web of Science, PubMed, Medline, and Google Scholar using a comprehensive search strategy incorporating keywords such as “astrocyte,” “glial cell,” “reprogramming,” “transdifferentiation,” “direct conversion,” “neuron,” “CNS disease,” “IS,” “Alzheimer,” “Parkinson,” “Huntington,” “ALS,” and “spinal cord injury.” Studies published between January 2015 and January 2025 were included to ensure the review reflects the most recent advancements in astrocyte reprogramming for CNS diseases. Eligible studies comprised original research and review articles focusing on the conversion of astrocytes into neurons while excluding non-English publications, duplicates, retracted articles, and studies lacking relevance to astrocyte reprogramming or CNS diseases. Priority was given to studies with well-defined methodologies and robust experimental designs to ensure scientific rigor and reliability.

## 3 ASTROCYTE PLASTICITY AND REPROGRAMMING POTENTIAL

Astrocytes, comprising 20-60% of the CNS cell population, are integral to brain homeostasis and pathology. Their tripartite synaptic structure enables them to regulate neuronal activity and influence cognitive processes such as learning and memory [16, 17]. Beyond physiological roles, astrocytes contribute to neurodegenerative and traumatic conditions, including PD, AD, and Huntington’s diseases (HD), as well as IS and CNS injuries [18, 19]. Positional identity in astrocytes is shaped by radial glial progenitor lineage and neuronal signaling [19, 20], enabling their involvement in synaptic development, pruning, and BBB maintenance. Their dual role in both exacerbating and mitigating CNS damage underscores their potential as therapeutic targets for neural repair [21].

The plasticity of astrocytes allows their transdifferentiation into neurons, a process driven by transcription factors (TFs) or small molecules. Pioneering work by Heins *et al*. demonstrated that *Pax6* expression induces neurogenesis in astrocytes *in vitro* [22], paving the way for strategies using *Neurog2*, *Ascl1*, or *Dlx2* to generate functional glutamatergic and GABAergic neurons [23, 24]. Notably, combinatorial expression of *Ascl1*, *Lmx1b*, and *Nurr1* reprograms astrocytes into dopaminergic neurons [25]. In *vivo* studies confirm astrocytes as the first non-neuronal cell type reprogrammable in the adult mouse brain [26, 27]. However, injury or neurodegeneration triggers reactive astrogliosis, marked by morphological and transcriptional shifts, which can lead to glial scar formation, a double-edged sword in post-injury recovery [28-30].

Advancements in gene delivery, particularly adeno-associated viruses (AAVs) paired with the human glial fibrillary acidic protein (hGFAP) promoter, enable precise astrocyte targeting *in vivo*. The hGFAP promoter drives robust gene expression in reactive astrocytes, silencing post-reprogramming to neurons [31-35]. This approach has shown therapeutic promise in IS, AD, and spinal cord injury (SCI) models, restoring neuronal populations and improving functional outcomes [31, 36, 37]. Despite progress, several challenges persist, including limited neuron yield under non-injury conditions and the protracted maturation of reprogrammed astrocytes into NeuN(+) neurons [14, 38, 39]. Dual signaling strategies, suppressing glial pathways while activating neuronal programs, enhance conversion efficiency and bypass immune rejection risk [40]. Collectively, astrocyte reprogramming represents a transformative strategy for CNS repair, bridging neuronal loss and functional recovery in neurodegenerative and injury contexts.

Among the various viral systems available for therapeutic gene delivery to the CNS, AAV currently represents the most suitable vector [41]. Astrocytes are widely distributed throughout the CNS [42, 43]. With a growing understanding of the factors that determine neuronal subtypes generated during astrocyte-to-neuron (AtN) transdifferentiation, AAV-mediated *in vivo* AtN conversion offers a promising approach for neuronal replacement in patients with brain injuries or neurodegenerative disorders [44].

Astrocyte reprogramming represents a groundbreaking therapeutic strategy for addressing neuronal loss in CNS diseases. The transformation of astrocytes into neurons may compensate for neuronal death and/or limit structural changes in tissues. Both mechanisms may be involved in the repair of impaired function under pathological conditions [45]. The conversion of reactive astrocytes into functional neurons provides a new strategy for neural repair. In IS models, this conversion can restore a portion of damaged neurons, significantly improving motor and cognitive functions [36]. In AD models, the transformation of astrocytes into neurons has been observed [31]. Similarly, in spinal cord injury, the efficient conversion of astrocytes has been demonstrated [37]. These findings lay an important foundation for the development of cell transformation-based neurorepair therapies.

Astrocytes possess a remarkable ability to transdifferentiate into neurons, a process that is regulated by dual signaling, involving the inhibition of glial activity signals while specifically activating neuronal phenotype pathways. As homologous cells of the nervous system, their innate immune compatibility effectively avoids the risk of transplant rejection. This transformation strategy not only overcomes the physical barrier of glial scar formation but also directly replenishes the neuronal loss in the damaged area [40].

## 4 OVERVIEW OF KEY RESEARCH ON REPROGRAMMING ASTROCYTES INTO NEURONS AS A POTENTIAL THERAPY

**Fig. (1):**
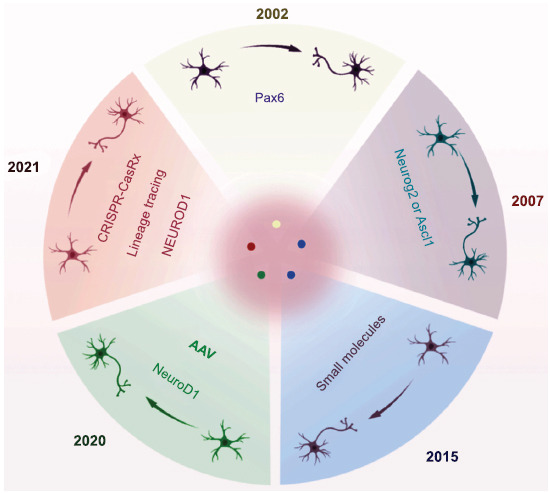
Key research on reprogramming astrocytes into neurons as a potential therapy.

In 2002, Heins *et al*. first demonstrated that retrovirus (RV) -mediated overexpression of *Pax6* could successfully induce neurogenesis in postnatal cortical astrocytes cultured in *vitro*, revealing the critical regulatory role of *Pax6* as an intrinsic fate determinant of the neurogenic potential of glial cells [22]. Subsequently, in 2007, studies found that single TFs (*e.g*., *Neurog2* and *Ascl1*) were also capable of reprogramming early postnatal cortical astrocytes, significantly inducing their conversion into functional neurons, thereby expanding the application of TFs in cell fate regulation [46]. In 2015, researchers utilized small molecule combinations to achieve the reprogramming of human astrocytes into functional neurons through epigenetic regulation. This process relied on the transcriptional activation of *NEUROD1* and *NEUROGENIN2*, with the reprogrammed neurons exhibiting long-term survival and functional integration both in *vitro* and in *vivo*, providing a novel strategy for chemical reprogramming [47]. In 2020, *NeuroD1*-mediated gene therapy successfully regenerated many functional neurons in ischemic injury models, significantly improving neuronal function and restoring motor and cognitive abilities, demonstrating its therapeutic potential in neural repair [14]. However, a 2021 study using lineage tracing revealed that neurons induced by AAV-mediated *NeuroD1* expression primarily originated from endogenous neurons rather than astrocytes. This finding not only emphasized the importance of lineage tracing in studying cell fate conversion in *vivo* but also highlighted the need to further optimize reprogramming strategies to achieve efficient AtN conversion (Fig. **1**) [48].

**Fig. (2):**
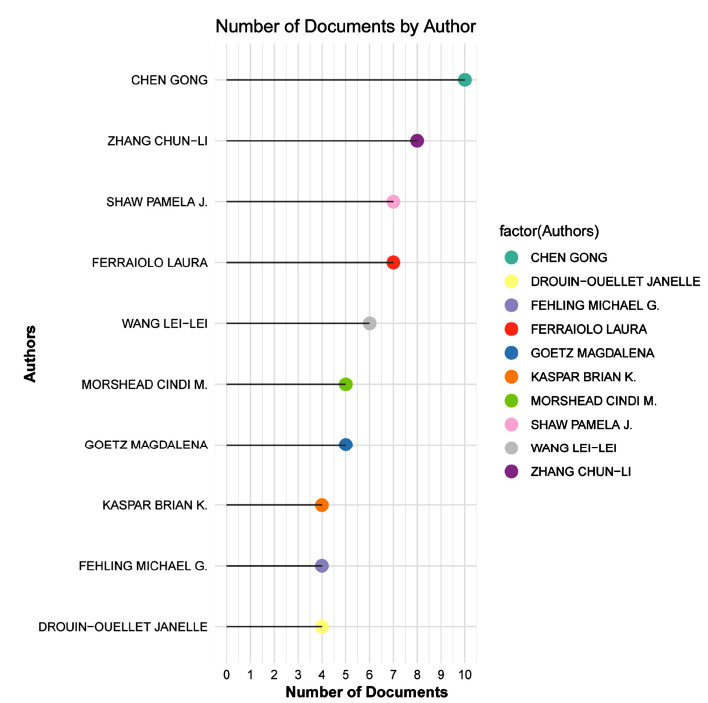
Top 10 authors with the highest number of publications on astrocyte-to-neuron (AtN) reprogramming in CNS diseases.

Over the past decade, researchers such as Chen Gong, Zhang Chun-Li, Shaw Pamela J., Ferraiolo Laura, and WANG Lei-Lei have published numerous high-impact studies in this field, advancing the clinical translation of astrocyte reprogramming technology (Fig. **2**). Among these, several highly cited studies have uncovered key mechanisms and therapeutic potentials. For instance, *in vivo* reprogramming of human and mouse astrocytes into induced dopaminergic neurons (iDANs) was achieved, with small molecules enhancing reprogramming efficiency by promoting chromatin remodeling and activating key signaling pathways. In a PD mouse model, NeAL218 successfully reprogrammed adult striatal astrocytes into excitatory iDANs, improving motor behavior and demonstrating potential clinical therapeutic prospects [49]. Rigorous lineage tracing methods revealed that the conversion of AtN in *vivo* primarily involved endogenous neurons rather than direct astrocyte-derived transformation, underscoring the importance of lineage tracing in cell fate conversion studies [48]. *NeuroD1*-mediated gene therapy effectively converted astrocytes into a large number of functional new neurons after ischemic injury, significantly restoring neuronal function and improving motor and cognitive abilities [14]. Similarly, AAV-mediated delivery of *NeuroD1* and *Dlx2* in an HD mouse model converted striatal astrocytes into GABAergic neurons, markedly improving motor function and extending lifespan, offering a potential disease-modifying therapeutic approach for HD and other neurodegenerative disorders [50]. A comprehensive review discussed how in *vivo* reprogramming technology, by converting endogenous glial cells into functional neurons, provides new avenues for central nervous system regeneration. Although clinical applications still face challenges, this technology holds promise for revolutionizing regenerative medicine by addressing issues such as immune rejection and functional integration (Table **1**) [51].

## 5 METHODS OF DIRECT ATN REPROGRAMMING

### 5.1 Transcription Factor-Mediated Astrocyte Reprogramming Technology

Glial cells in the brain can be directly reprogrammed into neurons without transitioning through a progenitor cell stage, a process known as direct neuronal reprogramming. This transformation can be achieved through the ectopic expression of a combination of neurogenic TFs or, in some cases, by a single TF. TFs are proteins that bind to specific DNA regulatory regions, modulating the rate of gene transcription. This binding can either upregulate or downregulate gene expression, thereby influencing protein production and altering cellular functions [52]. During nervous system development, neuronal-specific genes are gradually expressed, with several neuron-specific TFs playing pivotal roles. Overexpression of these TFs *via* vectors such as RV can modulate gene expression in non-neuronal cells, reprogramming them into neurons. Generally, strategies employing TF combinations have been reported to achieve higher levels of transcriptional control by promoting reprogramming at multiple checkpoints along the neurodevelopmental pathway. By carefully selecting TFs, this approach aims to enhance the efficiency of induced neuron (iNs) generation, potentially promoting functional recovery *in vivo* [53].

However, it is important to note that not all TFs drive reprogramming with equal efficacy. In some cases, adding more TFs to the mixture may impair the induction efficiency of iNs by reducing the likelihood of essential factors interacting with their targets [54]. Additionally, the context in which these TFs are expressed, whether in terms of cellular location or developmental stage, can significantly influence the outcome of the reprogramming process. For instance, ectopic expression of certain TFs, such as the MAF bZIP transcription factor A (MAFA), has been shown to block cellular differentiation when expressed outside its normal developmental environment [28]. These findings underscore the necessity of fine-tuning TF selection, dosage, and the timing of their application to optimize reprogramming outcomes.

Neurogenic TFs, including *NeuroD1*, *Ascl1*, *Brn2*, and *Myt1l*, are central to the AtN conversion process. For example, Guo *et al*. demonstrated that *NeuroD1* drives the transdifferentiation of cortical astrocytes and NG2 glial cells into functional glutamatergic and GABAergic neurons [31]. Notably, both *Neurog2* and *Ascl1* can reprogram AtN in the postnatal cortex, though *Ascl1* exhibits lower conversion efficiency compared to *Neurog2* [23, 24]. The *in vivo* direct reprogramming technique AtN has shown considerable promise for neural injury repair. In adult GFAP-Cre-Rosa-YFP mice, *NeuroD1* lentivirus-mediated reprogramming converted endogenous astrocytes in the peri-infarct area into mature neurons, restored cortical circuits and synaptic plasticity, and significantly improved motor, sensory-motor, and psychological functions, offering a potential strategy for IS recovery [55].

Moreover, *NeuroD1*-mediated *in vivo* reprogramming not only promotes the generation of new neurons but also facilitates their integration into the microcircuits of the visual cortex, resulting in direct visual responses and contributing to the restoration of visual function [56]. In an IS model of adult non-human primates, AAV-mediated *NeuroD1* gene therapy converted 90% of infected astrocytes into neurons, significantly increasing neuronal density, reducing microglia and macrophages, and protecting specific populations of inhibitory neurons. This approach demonstrated a broad therapeutic window and downregulation of *NeuroD1* post-neuronal maturation, highlighting its potential for brain tissue repair [57]. Similarly, *Ascl1* expression, both *in vitro* and *in vivo*, facilitates the transformation of mouse dorsal midbrain astrocytes into functional iNs, with highly efficient AtN conversion achieved in multiple brain regions of adult mice using a GFAP-AAV vector [32]. *NeuroD1*-mediated *in vivo* conversion has also regenerated a substantial number of functional neurons after ischemic injury, with AAV-based gene therapy not only replenishing lost neurons but also protecting damaged ones, leading to significant neurological recovery [14].

Despite these advances, challenges remain. For instance, while retroviral (RV) vectors expressing *Neurog2* and *Bcl-2* demonstrated relatively low efficiency in converting proliferating AtN in both young and aged mice following ischemic injury, optimization of viral vectors and TF combinations holds promise for improving conversion efficiency [58]. However, Wang *et al*. caution that high concentrations of viral vectors may lead to false-positive results and note that AAV-mediated overexpression of NeuroD1 does not specifically target astrocytes [48].

To address these limitations, Gleichman *et al*. developed a novel AAV vector that significantly improved astrocyte-specific expression by combining a 4x6T box structure with the GfaABC1D promoter, enhancing the specificity and efficiency of AtN technology [59]. AAV vectors are particularly advantageous for AtN induction because they are non-integrating, non-pathogenic, and capable of sustaining gene expression in both dividing and non-dividing cells, addressing key limitations of other delivery methods [60-62]. However, continuous expression of TFs may interfere with the maturation of reprogrammed neurons, potentially explaining the observed reduction in mature neurons during forced *Ascl1* expression [63]. Thus, while AAV vectors and TF combinations offer promising tools for AtN induction, careful regulation of TF expression is essential to ensure the successful maturation of reprogrammed neurons (Table **2**) [64].

### 5.2 miR-Induced Astrocyte Reprogramming Strategy

Precise regulation of gene expression within the CNS is critical during neurodevelopment, where a finely balanced interplay between neurogenic and anti-neurogenic genes establishes the necessary conditions for neuronal differentiation and maturation [65]. Among the key regulators of this process are miR, small non-coding RNAs ranging from 19 to 25 nucleotides in length. miRNAs play a pivotal role in neuronal reprogramming by binding to the 3' untranslated region (3'-UTR) of target mRNAs, leading to either translational repression or mRNA degradation [66]. One such miRNA, miR-124, is particularly significant due to its strong association with neurodevelopment. It is predominantly expressed in neurons, and its levels increase progressively during neuronal maturation [67, 68].

The functional importance of miR-124 in neuronal reprogramming has been demonstrated in several studies. Zheng *et al*. [69] demonstrated that a combination of miR-124 and three small molecules could induce the conversion of reactive astrocytes into multiple types of neurons in rats. miR-124 regulates astrocytic neuronal differentiation by targeting specific signaling pathways, such as the SOX9-NFIA-HES1 axis. Additionally, miR-124 regulates the expression of Ptbp1, a gene essential for neurogenesis. By modulating Ptbp1 levels, miR-124 further reinforces its role in promoting neuronal differentiation [70]. REST maintains non-neuronal transcripts by inhibiting miR-124a, and as REST leaves the miR-124a gene loci, it promotes the degradation of non-neuronal transcripts, enhancing the contrast between cell phenotypes [15]. Beyond miR-124, other miRNAs also contribute to the regulation of neurogenesis. Mo *et al*. demonstrated that miR-365 inhibits the expression of *PAX6* by targeting its 3'-UTR, thereby affecting the conversion of astrocytes into neurons [71]. miR-302/367 combined with VPA significantly improved spontaneous alternation and spatial memory in mice. Immunostaining showed a significant increase in NeuN labeling, and electrophysiological recordings indicated that induced neurons exhibited endogenous neuronal characteristics. The results suggest that reprogramming astrocytes into neurons *via* the miR-302/367 cluster could be a potential strategy for restoring learning and memory in AD patients [72]. However, downregulation of miR-365 promotes *PAX6*-mediated neurogenesis, suggesting a synergistic interaction between miRNAs and TFs [71]. Similarly, Rivetti *et al*. used miR-218 in combination with transcription factors to induce reprogramming of astrocytes into dopaminergic neurons both *in vitro* and *in vivo* [49]. Together, these findings underscore the complex and interconnected roles of miRNAs in modulating gene expression networks during neuronal reprogramming (Table **3**).

### 5.3 Small Molecule-Induced Astrocyte Reprogramming Strategy

Small molecule-based approaches have emerged as a pivotal strategy in neural repair and regeneration, offering distinct advantages over cell transplantation or viral vector-mediated gene therapy by circumventing risks such as tumorigenesis and immune rejection. These compounds modulate cellular signaling pathways to enable precise and controllable reprogramming processes, thereby presenting a promising therapeutic alternative [73].

Current studies demonstrate species-specific efficacy in AtN transdifferentiation. While small molecule cocktails effectively reprogram murine astrocytes into neurons, human astrocytes initially showed limited responsiveness [74]. This limitation has been partially overcome through optimized formulations containing forskolin, i-Bet151, and ISX-9, which successfully induced glutamatergic neurons from adult human astrocytes capable of surviving and maturing in murine brain environments [75]. Notably, Wu *et al*. [50] identified a chemical combination that preferentially generates cortical glutamatergic-like neurons from human astrocytes *in vitro*, mediated through dual mechanisms of neuronal TF activation and glial gene silencing [47]. Complementary findings by Fernandes *et al*. [76] revealed rapid astrocytic reprogramming in mice using a six-compound cocktail supplemented with neurotrophic factors (BDNF, GDNF, NT-3), yielding neurons with endogenous-like electrophysiological properties and regional specificity [77].

Furthermore, single-molecule interventions also demonstrate reprogramming potential. DAPT, a γ-secretase inhibitor targeting NOTCH signaling, facilitates the conversion of AtN *in vivo* with therapeutic implications for spinal cord injury [78]. Similarly, ginsenoside Rg1 induces cholinergic/dopaminergic differentiation through Notch/Stat3 pathway inhibition [79]. Functional validation of these chemically induced neurons has been achieved through mature culture systems and KCl-induced calcium flux assays, confirming neuronal activity and revealing neuroprotective effects, including oxidative stress reduction and enhanced survival [80].

Despite these advances, critical challenges persist in translating small molecule reprogramming to clinical applications. Key limitations include suboptimal *in vivo* delivery efficiency and transient cellular reprogramming stability. Emerging solutions incorporate nanotechnology to enhance drug distribution and targeting specificity [81] while leveraging the inherent reversibility of small molecule actions for dynamic regulation (Table **4**) [82].

In conclusion, chemical reprogramming represents a transformative approach to neural regeneration, eliminating risks associated with exogenous genetic material integration. Future research priorities should focus on: 1) elucidating molecular mechanisms underlying small molecule efficacy, 2) developing advanced delivery systems for *in vivo* applications, and 3) optimizing reprogramming protocols to enhance conversion efficiency and long-term stability. These advancements could establish new paradigms for treating neurological disorders through endogenous cellular reprogramming.

### 5.4 Direct Astrocyte-to-Neuron Reprogramming by Knockdown of Polypyrimidine Tract-binding Protein 1 (*PTBP1*)

The modulation of *PTBP1* expression has emerged as a critical strategy for inducing AtN. Genetic or pharmacological suppression of *PTBP1*, achieved through short hairpin RNA (shRNA), antisense oligonucleotides, or CRISPR-Cas13 systems, disrupts its dual roles in alternative splicing regulation and miR network coordination, thereby promoting neuronal phenotypic conversion [83-86]. Mechanistically, *PTBP1* maintains non-neuronal cellular identities by suppressing neuronal-specific splicing events, while its downregulation during neurodevelopment (mediated by miR-124 targeting) enables the accumulation of *PTBP2*, a paralog essential for neuronal differentiation.

Divergent findings highlight the complexity of *PTBP1*'s role in AtN. While Qian *et al*. [84] reported successful AtN conversion *via Ptbp1* knockdown, Wang *et al*. [48] observed no significant transdifferentiation *in vivo*, suggesting context-dependent regulatory mechanisms. This discrepancy underscores the need for systematic investigations into *PTBP1*'s interaction with ancillary factors, such as the neurogenic regulator *PAX6*, which modulates downstream targets essential for neuronal fate specification [87]. Notably, *PTBP1* suppression demonstrates therapeutic potential in PD models, where induced dopaminergic neurons integrate into striatal circuits, release stimulus-dependent dopamine, and ameliorate motor deficits, with these effects rigorously validated through chemogenetic (DREADD) approaches [84]. However, the generalizability of this conclusion requires further validation, as it assumes that AAV vectors can selectively and precisely target the intended cells, a level of specificity that may be challenging to achieve in practical applications.

Recent studies have also demonstrated that downregulation of *PTBP1* can successfully reprogram various types of glial cells, including oligodendrocytes, astrocytes, and Müller cells, into neurons [88]. Despite these advances, persistent controversies exist regarding *PTBP1*'s sufficiency for direct astrocytic transdifferentiation. Multiple studies report no AtN following *Ptbp1* deletion [34, 89].

## 6 THE MOLECULAR MECHANISM OF ASTROCYTE REPROGRAMMING INTO NEURONS

AtN reprogramming relies on two fundamental processes: (1) suppression of glial-specific gene expression and (2) activation of neurogenic programs driving neuronal conversion and differentiation. Advances in live-cell imaging, real-time quantitative PCR, and RNA sequencing have significantly enhanced our understanding of the molecular mechanisms underlying these processes. Central to this reprogramming is the dynamic regulation of epigenetic states, with the transcription factor *Sox2* playing a pivotal role. *Sox2* orchestrates the epigenetic landscape of neural progenitors by modulating the Polycomb Repressive Complex 2 (PRC2), thereby derepressing neurogenic fate determinants such as *Neurog2* and *NeuroD1*. This action establishes the epigenetic groundwork necessary for subsequent reprogramming events [90].

A critical aspect of *Sox2*-mediated reprogramming involves the transition of bivalent chromatin states into active configurations, a process essential for neurogenic gene expression. However, *Sox2* alone may be insufficient to achieve fully differentiated neuronal states, potentially due to inadequate activation of endogenous *Ascl1* in astrocytes [91]. While *Ascl1* functions as a transcriptional activator capable of accessing closed chromatin, its efficacy as a pioneer factor is limited. Key loci such as *NeuroD4*, regulated by *Ascl1* and *Neurog2*, are indispensable for successful reprogramming but remain subject to repression by the RE1-silencing transcription factor (REST) and H4K20me3 marks [92]. Strategies involving direct exogenous expression of these target genes or early removal of REST may circumvent these repressive barriers.

Transcriptional regulation and epigenetic factors are both critical in the molecular mechanisms of TFs. Notably, the heightened sensitivity of chromatin to the overexpression of TFs can lead to substantial changes in gene expression patterns. Several TFs, through forced expression of cytokines, are involved in the molecular mechanisms underlying neuronal reprogramming. For example, *Klf10*, *Myt1*, and *Myt11* regulate distinct phases of *Ascl1*-mediated reprogramming, from early neurogenesis to late electrophysiological maturation. Similarly, *Neurod4* and the chromatin remodeler Chd7 have been implicated in modulating reprogramming efficiency [1, 92-94]. Despite progress in characterizing transcriptional changes during reprogramming, significant gaps remain in understanding the epigenetic mechanisms governing cell fate conversion. Incomplete reprogramming of three-dimensional genome architecture and the regulatory roles of DNA methylation present unresolved challenges, necessitating advanced single-cell and multi-omics approaches to dissect these complex processes [92, 95-105].

Aging further complicates astrocyte reprogramming, as age-related alterations at the Ink4a/Arf locus correlate with diminished reprogramming potential. Notably, this capacity can be partially restored through *Dlx2* overexpression, implicating aging-associated molecular pathways as barriers to reprogramming [106]. Despite these challenges, astrocyte reprogramming offers distinct advantages for endogenous neuronal replacement therapies, including avoidance of immune rejection and enhanced functional integration of newly generated neurons. Additionally, metabolic reprogramming has emerged as a critical regulator of cell fate, with potential applications in promoting beneficial astrocytic transformations for IS recovery [107].

## 7 ASTROCYTE REPROGRAMMING AS AN EMERGING THERAPEUTIC APPROACH FOR NEURAL REPAIR IN CNS DISEASES

### 7.1 Ischemic Stroke (IS)

IS results in significant neuronal loss, but the reprogramming of astrocytes into neurons presents a promising therapeutic strategy for neural regeneration. Reactive astrocytes exhibit a neuroprotective phenotype, promoting vascular repair and aiding in the clearance of cellular debris and protein aggregates in IS models, thereby supporting motor function recovery [108]. Additionally, astrocytes can acquire progenitor-like characteristics through dedifferentiation, facilitating neuronal regeneration and repair [109-113]. Although astrocytes display stem cell-like characteristics under *in vitro* conditions, their capacity to transdifferentiate into neurons is limited *in vivo* [109, 111]. Under specific conditions, such as post-IS injury, reactive astrocytes can generate neurons *in situ* [114], highlighting their potential role in neural repair.

Research by Shen *et al*. demonstrated that in a middle cerebral artery occlusion model, astrocytes can transdifferentiate into neural stem cells following cerebral ischemia and ultimately mature into neurons [115]. This finding highlights the potential of astrocytes to contribute to neuronal regeneration under ischemic conditions. Similarly, in focal ischemia, the expression of *Neurog2* has been shown to enhance the ability of glial cells to convert into mature DCX-/NEUN+ iNs [116], further supporting the plasticity of astrocytes in response to injury. Yamashita *et al*. expanded on this concept by successfully inducing the transformation of post-IS astrocytes into neuronal cells through the ectopic expression of TFs *Ascl1*, *Sox2*, and *NeuroD1* in mice [115, 117]. These studies collectively underscore the role of key transcriptional regulators in driving AtN conversion.

Chen *et al*. confirmed that the expression of *NeuroD1 via* adeno-associated virus (AAV) could effectively convert astrocytes into functional neurons. These iNs not only formed synaptic connections with surrounding neurons but also exhibited functional electrophysiological properties [14], demonstrating their potential integration into existing neural circuits. Moreover, in adult mice, *in vivo,* chemical reprogramming of astrocytes into neurons using small molecules has been shown to be feasible. This approach not only replicates the expression of neuronal-specific markers, electrophysiological properties, and synaptic connectivity akin to endogenous neurons but also offers a promising avenue for developing neuronal replacement therapies [77].

In the context of IS therapy, the interactions between astrocytes and neurons play a pivotal role. Reprogramming strategies not only focus on the fate transition of astrocytes but also encompass their dynamic interactions with surrounding cells, which are critical for the maturation and functionality of the reprogrammed neurons [47, 48]. A deeper understanding of these intercellular communication mechanisms will be essential for optimizing reprogramming strategies, thereby enhancing neurorepair outcomes following IS.

### 7.2 Alzheimer’s Disease (AD)

AD is a progressive neurodegenerative disorder characterized by the pathological accumulation of amyloid-beta plaques and hyperphosphorylated tau proteins, which induce synaptic dysfunction and neuronal death. These pathological changes predominantly affect memory- and cognition-associated brain regions, including the hippocampus [118]. The pathogenesis of AD involves multifaceted mechanisms, such as neuroinflammation, immune activation, dysregulated lipid metabolism, impaired endoplasmic reticulum vesicle cycling, and defective autophagy [47].

Direct cellular reprogramming of terminally differentiated somatic cells into neurons has emerged as a promising therapeutic strategy for AD [119]. In AD model mice, cortical reactive glial cells were successfully reprogrammed into functional neurons *via* RV vector-mediated expression of the neurogenic transcription factor *NeuroD1* [31]. While *NeuroD1* demonstrated high efficiency in reprogramming human astrocytes *in vitro*, this approach failed to ameliorate cognitive deficits in AD mouse models, suggesting limitations in its translational therapeutic efficacy [31].

To address these limitations, alternative reprogramming strategies have been explored. Intracranial delivery of the miR-302/367 cluster into the hippocampal region of AD mice improved memory function, with transfected astrocytes exhibiting NeuN expression and induced neurons displaying electrophysiological properties comparable to endogenous neurons [72]. These findings highlight the potential of miRNA-based reprogramming as a complementary approach for neuronal replacement in AD therapeutics.

### 7.3 Parkinson's Disease (PD)

In addition to the loss of dopaminergic neurons, Parkinson's disease involves dysregulation of other neurotransmitters. The balance of neurotransmitters in the nigrostriatal pathway affects both motor and non-motor symptoms, with glutamatergic and GABAergic neurons regulating dopamine activity [15]. A recent study showed that manipulating the PTB/nPTB circuit reprogrammed astrocytes in the mouse midbrain and cortex into functional dopaminergic neurons [84]. This finding suggests a new therapeutic strategy for PD that goes beyond dopamine replacement.

Another study employed AAV9, a serotype capable of traversing the BBB, to vascularly deliver the *NeuroD1* transcription factor, facilitating the conversion of astrocytes into striatal neurons [38]. Recent studies using AAV-mediated delivery of *Neurog2* have shown efficient astrocyte-to-neuron conversion across multiple brain regions, including the midbrain and spinal cord. Approximately 60% of midbrain-derived neurons exhibited glutamatergic properties [120].

Giehrl-Schwab *et al*. (2022) found that GABAergic neurons generated by CRISPR-mediated reprogramming of striatal astrocytes could rescue motor function in a toxin-induced PD mouse model. This suggests a new therapeutic strategy for PD beyond dopamine replacement. The balance of neurotransmitter systems is crucial for effective PD treatment and remains an important area for further investigation [83].

### 7.4 Other CNS Diseases

Amyotrophic lateral sclerosis (ALS) is a neurodegenerative disease characterized by the loss of motor neurons in the CNS, leading to progressive paralysis and death [121]. Zhao *et al*. (2020) demonstrated that *SOD1*-mutant ALS mouse and human astrocytes can be reprogrammed into cells exhibiting motor neuron markers and electrophysiological properties through specific small molecule treatment [80]. Similarly, spinal cord injury (SCI), caused by trauma or progressive neurodegeneration, is a debilitating condition with a global annual incidence of 25-40 cases per million [122]. In the context of CNS repair, ectopic *SOX2* expression has been shown to reprogram glial cells into neurons, although the efficiency remains limited in the adult spinal cord [33, 91]. Wang *et al*. further revealed that downregulating the p53–p21 pathway enhances *SOX2*-mediated AtN reprogramming by overcoming the pathway's inhibitory effect on astrocyte-derived progenitor proliferation [34].

In HD, an autosomal dominant disorder, degeneration of GABAergic medium spiny neurons (MSNs) in the striatum and other brain regions leads to progressive motor, cognitive, and psychiatric symptoms. Recent studies have shown that AAV-mediated expression of *NeuroD1* and *Dlx2* can directly convert striatal astrocytes into functional MSNs. These reprogrammed neurons integrate into synaptic circuits, project to relevant brain regions, reduce striatal atrophy, improve motor function, and significantly extend the lifespan of R6/2 HD mice [50]. Together, these findings highlight the potential of cellular reprogramming strategies in addressing neurodegenerative diseases and CNS injuries.

## 8 LIMITATIONS AND PROSPECTS

The direct conversion of astrocytes into neurons presents a transformative approach for treating central nervous system CNS diseases. Yet, significant challenges must be addressed to advance this strategy toward clinical translation. Astrocytes exhibit dual roles in post-IS environments, engaging in both neuroprotective activities and maladaptive processes such as inflammation and glial scar formation, which may impede neuronal regeneration [123, 124]. While reactive astrocytes initially support tissue repair, their persistent activation can exacerbate neuronal damage, underscoring the need to balance their functional duality during reprogramming efforts. Current limitations in the efficiency and long-term stability of AtN conversion further highlight the necessity to elucidate the molecular mechanisms and regulatory networks governing this process. Enhancing the specificity and robustness of reprogramming will require deeper insights into epigenetic remodeling, transcriptional dynamics, and signaling pathways that drive cellular transdifferentiation [77].

A critical barrier to clinical application lies in defining rigorous criteria for evaluating the maturity of reprogrammed neurons. Although iNs exhibit neuronal markers, electrophysiological activity, and synaptic connectivity, transcriptomic analyses reveal substantial disparities between iNs and endogenous neurons [125]. Establishing standardized benchmarks for functional maturity, such as synaptic integration, neurotransmitter specificity, and network activity, will be essential to ensure clinical relevance. Concurrently, the functional integration of iNs into existing neural circuits remains poorly understood. Advanced lineage-tracing methodologies and real-time live-cell imaging technologies are needed to monitor dynamic morphological and functional changes during astrocyte-to-neuron conversion, providing mechanistic insights into synaptic rewiring and network incorporation [126]. Additionally, the role of thymidine analogs like EdU in tracking proliferative events during reprogramming requires careful validation to avoid confounding interpretations of cellular origins [64, 127, 128].

The potential mechanisms underlying the transdifferentiation of astrocytes remain largely unclear, particularly regarding the intrinsic cellular mechanisms, migration behaviors, and the relative contributions of microenvironmental signals. Differences between astrocyte subtypes may further influence reprogramming efficiency and the phenotype of the generated neurons. For instance, the choice of viral vector has a profound impact on the results: AAV induces a relatively mild inflammatory response compared to RV systems, but its efficiency is affected by promoter leakage and cell-type specificity limitations [129]. Research by Wang *et al*. shows that traditional Gfap promoters (*e.g*., GfaABC1D) drive non-targeted expression in neurons, whereas FLEX-AAV vectors enable specific targeting of astrocytes, thereby minimizing unintended neuronal conversion [48, 130]. Optimization of viral serotypes, promoter design, and delivery systems is thus imperative to enhance reprogramming precision.

Despite some progress, the current reprogramming efficiency remains suboptimal, with only a few reactive glial cells successfully converting into functional neurons. Furthermore, lineage tracing studies suggest that some so-called “astrocyte-derived” neurons may actually originate from other glial cell populations, which requires more rigorous validation [13, 48, 131, 132]. To translate cell reprogramming technology into clinical applications, it is essential to develop safe and effective therapeutic strategies. This includes optimizing reprogramming methods, such as using TFs, antisense oligonucleotides (ASOs), shRNA, small molecules, and CRISPR/Cas systems, to minimize harmful effects and enhance the efficiency, survival, and integration of iNs *in vivo*. Additionally, it is crucial to reduce the potential risks associated with viral vectors and to develop controlled release and precise targeting technologies. Furthermore, it is important to further investigate the efficiency differences in reprogramming non-neuronal cells into iNs to improve the universality and applicability of this approach [133, 134].

Future research on astrocyte reprogramming must adopt diverse approaches to address key challenges and advance therapeutic applications. A critical priority is to decipher the molecular mechanisms of reprogramming using technologies such as single-cell transcriptomics, live-cell imaging, CRISPR-Cas9 gene editing, and/or optogenetics, particularly to uncover how epigenetic and transcriptional cascades drive AtN conversion [135-137]. Concurrently, optimizing delivery systems is essential, including the development of cell-specific targeting technologies, enhancement of viral vector tropism, and exploration of intelligent drug delivery strategies such as microbubbles for sonoporation and gold nanoparticles [138, 139]. Equally important is understanding the cellular and molecular mechanisms by which the local microenvironment guides the transdifferentiation of glial cells into neurons [140].

To ensure clinical relevance, standardized evaluation metrics must be established, encompassing benchmarks for *in vivo* iNs maturity, functionality, and safety. Finally, integrating reprogramming strategies with complementary therapies, such as neuroprotective agents and anti-inflammatory treatments, could synergistically enhance neuronal survival, connectivity, and functional recovery. These efforts aim to bridge the gap between experimental findings and clinical translation, paving the way for effective neural repair in CNS disorders.

## CONCLUSION

Despite the challenges, the potential application of direct AtN reprogramming in CNS disease treatment remains promising. Using specific TFs, small molecules, and microRNAs, astrocytes can be effectively reprogrammed into neurons, offering a new direction for CNS diseases' neurological recovery. As reprogramming techniques continue to evolve and improve, direct AtN reprogramming may become a viable strategy for CNS disease treatment, providing renewed hope for patients.

## AUTHORS’ CONTRIBUTIONS

The authors confirm their contribution to the paper as follows: study conception and design: RQ; draft manuscript: X. Lai, WX, QQ, X. Liang, MX, LC. All authors reviewed the results and approved the final version of the manuscript.

## Figures and Tables

**Table 1:** The top five most-cited papers on astrocyte reprogramming into neurons.

**Paper**	**DOI**	**Total Citations**	**TC per Year**	**Normalized TC**
Cervo Pia Rivetti Di Val, (2017)	10.1038/nbt.3835	246	27.33	6.06
Wang Lei-Lei, (2021)	10.1016/j.cell.2021.09.005	186	37.2	7.69
Chen Yu-Chen, (2020)	10.1016/j.ymthe.2019.09.003	174	29	4
Wu Zheng, (2020)	10.1038/s41467-020-14855-3	126	21	2.9
Li Hedong, (2016)	10.1016/j.neuron.2016.08.004	116	11.6	2.59

**Table 2:** Summary of transcription factors (TFs) for inducing astrocytes to different neuronal subtypes and conversion efficiency.

**Reprogramming Factors**	**Methods**	**Source**	**Efficiency of Reprogramming**	**References**
*NeuroD1*	RV	Mouse	90% NeuN+ at day 7	[31]
*NeuroD1*	RV	Human	90% NeuN+ at day 5	[31]
*Ascl1*	RV	Mouse	51% TuJ1+ after day 12	[23]
*Neurog2*	RV	Mouse	>85% TuJ1+ after day 12	[23]
*NeuroD1*	LV	Mouse	66 % NeuN+ at 4 weeks	[55]
*NeuroD1*	AAV	Mouse	>50 % NeuN+ at 3 weeks	[56]
*NeuroD1*	AAV	Monkey	52.6 % NeuN+ at 4 weeks	[57]
*Ascl1*	AAV	Mouse	64.4 ± 3.4 % NeuN+at day 30 (striatum)	[32]
*Ascl1*	AAV	Mouse	93.9 ± 1.2 % NeuN+at day 30 (cortical)	[32]
*NeuroD1*	AAV	Mouse	~ 70% NeuN+ at day 17	[14]
*NeuroD1*	AAV	Mouse	~ 48% NeuN+ at day 135	[36]
*Neurog2*	RV	Mouse	Very low, ~1%	[58]
*Neurog2+Nurr1*	AAV	Mouse	~ 53% NeuN+ at day 24	[64]

**Abbreviations**: AAV, Adeno-associated virus; LV, lentiviral.

**Table 3:** Summary of miRNA for inducing astrocytes to different neuronal subtypes and conversion efficiency.

**Reprogramming Factors**	**Methods**	**Source**	**Efficiency of Reprogramming**	**References**
miR 124 + 3C	Mimic	Rat	64.4 % Tuj1+ + and 87.3 % NeuN+ at day 7	[69]
miR 124 + ISX9	LV	Mouse	71.8 % NeuN+ at 3 weeks	[70]
miR 124 + ISX9	AAV	Mouse	51.6 % NeuN+ at 3 weerks, 79.4 % NeuN+ at 8 weeks	[70]
miR 124	Lipo.2000	Mouse	~35 % Tuj1+at day 7	[70]
miR 124 + ISX9	Lipo.2000	Mouse	62 % Tuj1+ at day 7	[70]
miR-302/367+VPA	LV	Mouse	NeuN+ at 2 weeks	[72]

**Abbreviations**: AAV, Adeno-associated virus; LV, lentiviral; ISX9, isoxazole-9.

**Table 4:** Summary of small molecules for inducing astrocytes to different neuronal subtypes and conversion efficiency.

**Small Molecules**	**Source**	**Efficiency of Reprogramming**	**References**
DFICBY	Mouse	11.3 % NeuN+(striatum) 10.7 % NeuN+(cortex) *in vivo*	[74]
DFICBY	Mouse	88.2% Tuj1+ at day 16 *in vitro*	[74]
VCR +(forskolin, iBet151, and ISX-9)	Human	70% VGULT1+ at day 40	[74]
SLCD	Human	78% VGULT1+	[75]
LDN193189, SB431542, TTNPB, Tzv, CHIR99021, VPA, DAPT, SAG, Purmo	Human	68.7% ± 4.2% NeuN+ at day 8, 88.3% ± 4% vGLUT1+ at 2 months, 8.2% ± 1.5% GAD67+ at 2 months	[47]
LDN193189, SB431542, TTNPB, thiazovivin, CHIR99021, VPA, DAPT, SAG, and purmorphamine	Human	97.1 ± 1.1% FoxG1+; 71.4 ± 3% Ctip2+;86.4 ± 3.4% Tbr1+	[47]
6C-NIMVPA, CHIR99021, Repsox	Mouse	82 ± 6 % Tuj1+ at day 4	[76]
	Mouse	32% DCX+ at day 12.24% NeuN+ at day 18	[77]
Ginsenoside Rg1	Rat	26.0 ± 1.5 % Tuj1+13.6 ± 0.8%; MAP2+, 22.2 ± 1.1% NeuN+ at day 7	[79]
KFYPR	Mouse	91.9 ± 1.6% Tuj1+	[80]
KFYPR	Human	93.7 ± 1.6% Tuj1+ at 2 weeks	[80]

## LIST OF ABBREVIATIONS

AAVs: Adeno-associated Viruses
AD: Alzheimer’s Disease
ALS: Amyotrophic Lateral Sclerosis
ASOs: Antisense Oligonucleotides
AtN: Astrocyte-to-neuron
BBB: Blood-brain Barrier
CNS: Central Nervous System
hGFAP: Human Glial Fibrillary Acidic Protein
iDANs: Induced Dopaminergic Neurons
iNs: Induced Neurons
IS: Ischemic Stroke
miR: MicroRNA
MSNs: Medium Spiny Neurons
PD: Parkinson’s Disease
PTBP1: Polypyrimidine Tract-binding Protein 1
REST: RE1-silencing Transcription Factor
RV: Retroviral
SCI: Spinal Cord Injury
ShRNA: Short Hairpin RNA
TFs: Transcription Factors
3'-UTR: 3'untranslated Region
